# How do residents respond to uncertainty with peers and supervisors in multidisciplinary teams? Insights from simulations with epistemic fidelity

**DOI:** 10.1186/s41077-024-00281-8

**Published:** 2024-02-12

**Authors:** Sarah Blissett, Jamila Skinner, Harrison Banner, Sayra Cristancho, Taryn Taylor

**Affiliations:** 1 Division of Cardiology, Department of Medicine, Schulich School of Medicine and Dentistry, Western University, London, ON Canada; 2 Centre for Education Research and Innovation, Schulich School of Medicine and Dentistry, Western University, London, ON Canada; 3 Schulich School of Medicine and Dentistry, London, ON Canada; 4 Department of Obstetrics and Gynecology, Schulich School of Medicine and Dentistry, London, ON Canada; 5 Department of Surgery, Schulich School of Medicine & Dentistry, Western University, London, ON Canada

**Keywords:** Uncertainty, Simulation, Multidisciplinary teams

## Abstract

**Background:**

Residents struggle to express clinical uncertainty, often exhibiting negative cognitive, behavioral, and emotional responses to uncertainty when engaging with patients or supervisors. However, the Integrative Model of Uncertainty Tolerance posits that individuals may have positive or negative responses to perceived uncertainty. Situational characteristics, such as interactions with other health professionals, can impact whether the response is positive or negative. The team context in which residents interact with resident peers and supervisors could represent varying situational characteristics that enable a spectrum of responses to uncertainty. Understanding the situational characteristics of multidisciplinary teams that allow residents to display positive responses to perceived uncertainty could inform strategies to foster positive responses to uncertainty in other contexts. We explored resident responses to perceived uncertainty in a simulated multidisciplinary team context.

**Methods:**

A simulation-primed qualitative inquiry approach was used. Fourteen residents from Cardiology and Obstetrics and Gynecology participated in simulation scenarios involving pregnant patients with heart disease. We incorporated epistemic fidelity through the deliberate inclusion of ambiguity and complexity to prompt uncertainty. Audio recordings of debriefing sessions were analyzed using directed content analysis.

**Results:**

Residents recognized that uncertainty is unavoidable, and positive responses to uncertainty are crucial to team dynamics and patient safety. While residents had positive responses to expressing uncertainty to peers, they had predominantly negative responses to expressing uncertainty to supervisors. Predominant negative response to supervisors related to judgement from supervisors, and impacts on perceived trustworthiness or independence. Although residents recognized expressing uncertainty to a supervisor could identify opportunities for learning and resolve their uncertainty, the negative responses overshadowed the positive responses. Residents highly valued instances in which supervisors were forthcoming about their own uncertainty.

**Conclusions:**

Through participation in simulations with epistemic fidelity, residents reflected on how they perceive and respond to uncertainty in multidisciplinary teams. Our findings emphasize the role of situational characteristics, particularly peers and supervisors, in moderating responses to perceived uncertainty. The productive discussions around responses to uncertainty in debriefing sessions suggest further studies of multidisciplinary simulations could enhance our understanding of how uncertainty is expressed, and potentially be used as an instructional intervention to promote positive responses to uncertainty.

## Background

Uncertainty is pervasive in clinical medicine. While some uncertainty can be resolved by addressing knowledge gaps, irreducible uncertainty relating to diagnosis and management is unavoidable [1]. As the paradigm around uncertainty has shifted from minimizing uncertainty to tolerating uncertainty [2], expressing uncertainty in clinical environments can shift from becoming hushed to heard. Yet, educators face a problem: residents often struggle to express their clinical uncertainty.

The Integrative Model of Uncertainty Tolerance provides a framework to situate how residents express uncertainty [2, 3]. Within this model, probability, ambiguity, and complexity are stimuli that can lead to a perception of uncertainty. If uncertainty is perceived, then it can lead to cognitive, behavioral, and emotional responses. These responses could have negative or positive valences. For example, a negative cognitive response may involve feeling threatened when uncertainty is perceived, whereas a positive cognitive response could be conceptualizing the uncertainty as an opportunity to learn. The perception of uncertainty and response to uncertainty is moderated by stimulus characteristics, individual characteristics, situational characteristics, cultural factors, and social factors.

These moderating factors have relevance to how residents perceive and respond to uncertainty in the various clinical learning environments they encounter during training. Situational characteristics refer to time, support, and communication with patients and others [2]. An important situational characteristic in considering how residents respond to uncertainty in clinical contexts is the other participants involved. Our current understanding of how residents express uncertainty is informed by previous studies that explored how residents navigate uncertainty in one-on-one settings with patients [4] and with supervisors from their own specialty [5, 6]. The term supervisor refers to the supervising physician, which is synonymous with attending physician, staff physician, or consultant. Residents experience challenges with expressing uncertainty to patients and supervisors including determining the stimulus of their uncertainty [4, 5], deciding when and when not to express uncertainty given the potential for perceived cognitive (threatened sense of self), behavioral (avoidance, loss of procedural opportunities), and emotional (fear of loss of trust) responses [2, 6]. Residents gravitate to negative emotional, cognitive, and behavioral responses to uncertainty in scenarios involving patients and supervisors [3].

Initial explorations of how residents navigate uncertainty with other residents from their own specialty in clinical settings identified that junior residents engaged with senior residents to validate their uncertainty through member-checking [6]. In this situation where residents from the same specialty worked together in non-simulated settings, residents had positive cognitive and behavioral responses to uncertainty, contrasting the negative responses to uncertainty with supervisors from the same specialty.

However, these data do not capture the entire spectrum of clinical contexts in which residents encounter uncertainty. Residents often work in multidisciplinary teams comprised of residents and supervisors from other specialties and other health professionals when caring for patients. Despite the critical importance of resident responses to uncertainty in multidisciplinary teams on team function, how residents might respond to uncertainty when working in multidisciplinary teams with residents and supervisors from other specialties has not yet been studied.

Specialty-specific elements may prompt different responses amongst residents from different specialties. The degree of hierarchy between residents and supervisors [7–9] differs amongst specialties. Surgical specialties are described as having more individualism, conferring a vertical structure with the supervising surgeon leading the decision-making process. In this structure, one’s position within the hierarchy determines the voice they have within the team [10]. Consequently, there is less opportunity for input from residents [9] and the perceived stakes of expressing uncertainty are high. In medical specialties, a more collective approach between trainees and supervisors has been described, where all team members are encouraged to participate [9]. Additionally, surgical trainees reported that expressing uncertainty can lead to fewer procedural or surgical opportunities [11], a consequence that is less likely to be experienced by trainees in medical specialties. These different perspectives may inform how residents from different specialties respond to uncertainty.

If educators and supervisors are to support residents in overcoming the struggles they have with expressing uncertainty, then we must further understand how residents respond when faced with clinical uncertainty in contexts beyond one-on-one interactions with patients or supervisors. Educators and supervisors need to understand how residents respond to uncertainty in multidisciplinary teams, with members from within and outside their own specialty. This novel study uses simulation-primed inquiry [12] to explore resident responses to perceiving uncertainty in the varying situational characteristics of a simulated high-fidelity multidisciplinary team context.

## Methodology

### Design

We used a simulation-primed qualitative inquiry approach [12] to explore the socially situated phenomenon of uncertainty in multidisciplinary teams. Simulation-primed qualitative inquiry involves three steps (1) determining applicability, with this design most suited to explore complex, context-bound topics with multiple perspectives; (2) designing the simulation, including incorporating elements to prime participants; and (3) planning data collection, and including creation of a semi-structured interview guide and attention to psychological safety [12]. In this approach, simulation scenarios are deliberately designed to prime participants to reflect on a topic in the debriefing sessions, rather than using the simulation itself as an intervention. In our case, we designed the simulation scenarios to prompt uncertainty and used the debriefing sessions to explore perceptions around responses to uncertainty in multidisciplinary teams in the simulation scenario and also in prior clinical encounters.

### Participants

We invited senior obstetrics and gynecology (OBGYN) (postgraduate year [PGY] 4 and 5) and Cardiology residents (PGY 4–6) at Western University to participate in simulation scenarios that would usually be managed by senior trainees in the clinical workplace. We included senior trainees from Cardiology and OBGYN to facilitate uncertainty that was not solely attributed to knowledge gaps. OBGYN nurses were full participants in the simulation scenarios to maintain realism, though they were not included in the research study.

### Simulations

The simulations involved managing a pregnant patient with acquired heart disease, presenting with acute cardiac symptoms (chest pain and dyspnea) and hemodynamic instability in the third trimester of pregnancy (Table 1). The simulated patients were not in labor during the simulation. This content domain was chosen given the inherent need for multidisciplinary care from OBGYN and Cardiology and the known limited exposure to pregnant patients with heart disease during training [13, 14]. All simulations were conducted in a high-fidelity simulation centre, using SimMOM (Laederal Medical). SimMOM is a high-fidelity mannequin with vital signs, heart sounds, lung sounds, pulses, intravenous access, and fetal heart rate monitoring amongst other features. SimMOM’s voice was provided by an actor in the control room, which allowed for real-time responses to questions and therapeutic interventions.

**Table 1:** Overview of simulation scenarios

	Scenario 1: Antepartum endocarditis	Scenario 2: Antepartum spontaneous coronary artery dissection
Summary	Hypotension secondary to sepsis due to tricuspid valve endocarditis with septic emboli and gram-positive bacteremia in a person who uses intravenous drugs	Pregnant patient with ischemic chest pain, anterior T-wave inversions, and positive troponin at 34 weeks. The patient developed ventricular fibrillation and a cardiac arrest. The scenario concludes 4 min after the cardiac arrest.
Presenting information	Becky Jones is a 36-year-old G5 P4 woman who estimates she is about 36 weeks GA. She presented with total body pain, non-productive cough and a fever that has been ongoing for the last day. No known COVID-positive contacts. Lives in shared housing. HR 110, 89/40 mmHg, RR 22, O2 sat 97% room air, temp 38.5 Fetal heart rate: 150 with moderate variability and no decelerations.	Stephanie Barrow is a 34-year-old G1 P0 woman at 34 weeks GA. She developed crushing, retrosternal chest pain radiating to the left arm about 2 h prior to presentation. Her partner drove her to OB triage for further evaluation. She arrives at OB triage at 1 am. HR 100, 135/70 mmHg (equal in both extremities), RR 22, O2 sat 99% room air, temp 36.5 FHR 150 with moderate variability and no decelerations.
Synopsis of Scenario	Further history reveals current use of cocaine; no features of active labour. Physical exam demonstrates closed cervix and holosystolic murmur at the left lower sternal border. Investigations released upon request from the treating team reveal leukocytosis, elevated creatinine, elevated lactate, toxicology positive for cocaine, negative rapid COVID test, ECG demonstrating sinus tachycardia, chest X-ray demonstrating a right lower lobe infiltrate, and POCUS images demonstrating a large vegetation on the tricuspid valve with severe tricuspid regurgitation. Initial management includes initiation of intravenous fluidsbroad-spectrum antibiotics including MRSA coverage, consultation from infectious disease and/or cardiac surgery. If fluids are not given, the patient becomes progressively more hypotensive.	Further history reveals no features concerning pulmonary embolism or neurologic symptoms. Physical exam reveals closed cervix and normal cardiovascular exam. Investigations released upon request reveal abnormal ECG with deep anterior T wave inversions. Expected initial management includes aspirin, initiation of heparin, initiation of nitroglycerin, and consideration of a second antiplatelet agent and coronary angiogram when the pain does not improve with nitroglycerin. The patient becomes unresponsive with rhythm revealing ventricular fibrillation, necessitating cardiopulmonary resuscitation with modifications for pregnant patients including lateral displacement of the uterus, early intubation, and consideration of resuscitative hysterotomy.
Uncertainty prompts	*Patient statements:* Patient asks: “What is going on?” “Do I need surgery on my heart?” “Can you make me feel better?” “What if I go into labour- do I need a C-section?” *Complexity around diagnosis and management:* Patient initially denies drug use Presence of murmur and intravenous drug use added complexity to the presentation of sepsis and symptoms of pneumonia *Ambiguity around diagnosis and management:* Investigations are only released when requested ECG, chest X-ray, and POCUS images needed to be interpreted by the treating team, no interpretation was provided with these tests.	*Patient statements:* Patient asks: “Why are you calling a cardiologist?” “Are these medications safe during pregnancy?” *Complexity around diagnosis and management:* Scenario occurs overnight CT angiogram not available if requested to assess for pulmonary embolism *Ambiguity around diagnosis and management:* Investigations are only released when requested ECG findings could be due to anterior ischemia or pulmonary embolism, requiring prioritization of diagnosis based on clinical features. Management of spontaneous coronary artery dissection is ambiguous with the role of a second antiplatelet agent and angiography in a non-ST elevation myocardial infarction context debated.

Two simulation scenarios were developed with input from simulation and content experts in OBGYN and Cardiology: (1) an antepartum presentation of endocarditis presenting with fever, dyspnea, and chest pain and (2) an antepartum presentation of spontaneous coronary artery dissection presenting with chest pain (Table 1). Complementary information included lab values, chest X-rays, ECGs, point-of-care echocardiography (POCUS) images, and fetal heart rate tracings. Teams were required to make urgent decisions about diagnosis and management in the scenarios. We incorporated stimuli for uncertainty in both cases to align with the recent focus on epistemic fidelity in simulations to reflect the complexity and ambiguity of authentic clinical practice [15]. We accomplished this through the deliberate inclusion of (a) probability statements from the patient, (b) complexity around the diagnosis and management, and (c) ambiguity around the diagnosis and management. Each resident participated in both scenarios.

### Protocol

In each simulation, residents from OBGYN and Cardiology worked together with an OBGYN nurse to provide care for a pregnant patient with heart disease. Simulations were anticipated to take 30 min and were stopped when learning objectives were achieved. The confederate OBGYN or Cardiology supervising physician would join the simulation if called by the resident.

Immediately following each simulation scenario, all participants attended a debriefing session using the PEARLS framework [16]. Debriefing sessions were facilitated by SB and HB, both with expertise in caring for patients with heart disease during pregnancy and medical simulation education. Clarifying questions regarding how trainees expressed uncertainty were incorporated into the debriefing sessions, e.g., “Were there any aspects of the case that you were unsure about?”, “Can you tell us about how you decide to say ‘I don’t know’?” Participants reflected *on* their performance (i.e., what they did in the moment and why) and *beyond* their performance (i.e., how they would usually approach such a scenario and why). Each debriefing session was audio recorded and professionally transcribed verbatim.

### Analysis

We focused our analysis on the resident participants given the supervisors participating in the study were confederates. The qualitative analysis was based on debriefing sessions, and not any of the conversations from the simulation scenarios, in keeping with the simulation-primed methodology.

Data analysis occurred after data collection was complete given the design of our study. We analyzed audio recordings of debriefing sessions using an inductive, directed content analysis [17]. JS and SB independently coded each transcript line by line. After all debriefing sessions were coded, JS and SB met to identify themes relating to how residents responded to uncertainty from the codes. Themes and codes were then further refined through subsequent analytic meetings with all members of the research team. We used the Integrated Model of Uncertainty Tolerance [2, 3] to classify the valence (positive or negative) and types (cognitive, behavioral, or emotional) of responses to uncertainty by individual residents. We also noted the situational characteristics of the team context (e.g., peers vs. supervisors) that moderated responses to uncertainty.

### Reflexivity

Our research team consisting of a medical trainee (JS), specialists from both OBGYN (TT, HB) and Cardiology (SB), and medical educators (TT, HB, SB, SC) allowed for various experiences with uncertainty to inform the analysis. The author team shared common perspectives on uncertainty that informed the analytic lens: (1) uncertainty is unavoidable in clinical contexts, (2) expressing uncertainty is acceptable to patients, peers, and supervisors, and (3) expressing uncertainty should ideally be encouraged by all levels of trainees and faculty. We were mindful that the author team was predominantly supervisors, and findings would accordingly be viewed from the lens of identifying what supervisors should know about how trainees respond to uncertainty. We remained attentive to identifying discrepant perspectives during the analysis.

### Ethics approval

The project received ethics approval from Western University Research Ethics (REB #118181).

## Results

Table 2 summarizes the demographics of the 14 participants and the team composition. The results section is structured around our four main findings, with findings relating to either experience within the simulations or prior clinical encounters. Verbatim quotations are provided to illustrate our findings, using anonymous participant codes (OBGYN# for OBGYN residents and Cardio# for cardiology residents).

**Table 2:** Participant demographics and team composition

Gender	
Male Female	6 8
Training level	
PGY 4 PGY 5 PGY 6	7 5 2
Specialty	
OBGYN Cardiology	6 8
Team composition	
1 Cardio+OBGYN	10
1 Cardio+OBGYN	2

*PGY* postgraduate year, *OBGYN* obstetrics and gynecology

### Realities and ideals of uncertainty in multidisciplinary teams

Residents appreciated that irreducible uncertainty is unavoidable in clinical activities:

“I feel like we're dealing with uncertainty all the time... In medicine, it's a game of probability” (Cardio 7).

Residents reflected that they encounter scenarios that do not necessarily have a defined answer, and valued team discussions to provide care for patients in the face of uncertainty:

“My sense was that with a case like this, it would usually be like what we would get our staff involved, but [there] would also be like a multidisciplinary discussion regarding decision making.” (OBGYN, 4)

“I don't think there is a definite answer to the situation that someone with endocarditis with septic emboli would go for surgery. I think as you mentioned, it would be a team discussion amongst the patient, especially the patient, if she would ever want surgery.” (Cardio, 4)

Residents valued expressing uncertainty in a manner that could be easily recognized and understood by others. Residents appreciated that there could be detrimental consequences to team decisions if uncertainty is not communicated or recognized:

“It would behoove all of us to be more open about expressing our various uncertainties about patient care. Because it can cue the other team members to your uncertainty….Whereas when people run around expressing confidence, then things sometimes move a little bit too quickly” (Cardio 8)

### Resident responses to uncertainty with peers in multidisciplinary teams

Residents described positive cognitive and emotional responses to expressing uncertainty with peers in the simulation scenario and in their prior clinical encounters. They indicated ease in expressing uncertainty to residents from other specialties and felt comfortable in doing so. They prioritized clear communication to ensure the other specialties understood that they needed their help with the patient. They did not perceive fears of judgment or loss of trust from expressing uncertainty to their peers:

“I don't mind telling other residents whether I know what's going on or not”. (OBGYN 6, in reference to residents from other specialties)

“I think I'm pretty forthcoming about uncertainty with my colleagues.... I'm pretty comfortable with that. I feel like it's better than pretending you know what you're talking about when you don’t” (OBGYN 3)

Residents communicated their uncertainty using direct and indirect terms. When reflecting on communicating uncertainty using the expression “I don’t know” to a resident from another specialty, as was observed in a simulation scenario, residents expressed that some may perceive it to be unprofessional from the patient’s perspective, but prioritized honest expressions:

“I mean, it's a little unprofessional maybe, but at least it’s honest.” (OBGYN 3)

Residents often expressed uncertainty indirectly to their peers, in ways that allowed them to communicate their uncertainty without exposing their uncertainty to the simulated patient. They asked clarifying questions “what [blood pressure] do you usually target?” (Cardio 3), deferred questions to the other specialty “what would be your preference from an OB standpoint?” (Cardio 3), and made qualifying statements about their interpretations “I just saw some T wave inversions and I wanted you to look over [the ECG]” (OBGYN 2)

Their openness to express uncertainty related to defining the content of the uncertainty as out of their self-defined scope of practice. They expressed that the perceived out-of-scope content could be managed by team members from the other specialties:

“With regards to the ECG, I just thought peaked T’s and I couldn’t really tell what was going on either way, but I knew it wasn’t an OB [issue] (OBGYN 1)

“Except when it’s OB stuff and it’s like we don’t really know what that is” (Cardio 1)

### Resident responses to uncertainty with supervisors

Although residents valued expressing uncertainty, they had negative cognitive and behavioral responses to expressing uncertainty to supervisors. Expressing uncertainty to their supervisor could be perceived as a potential weakness, associated with threats to judgement and trustworthiness:

“As a senior resident, if you're saying you don't know what is going on and like the consultants like, oh no, there is actually a clear picture here and you missed it. I feel like that reflects poorly. So there's a bit more hesitancy saying you don't know what's going on when you're talking to the consultant, whereas versus when you're talking to the patient... just as a senior learner, where you're being evaluated on every interaction, I find there’s just a little bit more of a hesitation” (OBGYN 6).

Residents desired to have a complete understanding of the patient’s presentation prior to discussing it with supervisors. They perceived that supervisors would judge them if they did not have a complete understanding of the presenting diagnosis and a finalized management plan, ideally preferring to avoid expressing uncertainty:

“I do feel a bit more responsible to come up with a picture that's clear and concise and directional when I'm talking to my consultant…Whereas when I go to the consultant, there's like an expectation that I'm like approaching consultant level and I should be able to like, run this by myself.” (OBGYN 6)

“You have to work through, I feel like, to get enough investigations and have at least a bigger, or more clear type differential in mind before calling them, and I usually almost always call with a plan” (Cardio 1)

However, similar to how residents responded to uncertainty with peers, residents adopted positive cognitive responses to uncertainty with their supervisors when they perceived the content was out of their scope of practice:

“I think because it’s a pregnant person, I think would probably call earlier…. And we typically don't involve the [supervisor] until we kind of have a relatively clear picture” (Cardio 3)

While residents gravitated toward negative responses to uncertainty when communicating with supervisors in general, they acknowledged that the supervisor recognizing a learner was uncertain could lead to positive behavioral responses in ensuring patient safety. Residents indicated they were more likely to express uncertainty if they were managing a young patient or an acutely ill patient, where they perceived the benefits of expressing their uncertainty outweighed perceived judgement from supervisors:

“The younger the patient the more likely I am to call a [supervisor] for something” (Cardio 1).

“Oh yes, [the supervisors] definitely get mad. But I feel like it’s a sick patient and I think that the [supervisor] needs to know when there’s a sick patient.” (OBGYN 1)

Residents also described positive cognitive and emotional responses in recognizing that expressing uncertainty provided an opportunity for supervisors to resolve the uncertainty, prompted teaching from supervisors, and provided an opportunity to cultivate curiosity:

“So, you [supervisors] know everything. So, you tell me” (OBGYN 2)

“Our [supervisors] are pretty good about teaching us and identifying those gaps in our knowledge. But they're even better at that when we tell them we don't know. And when we ask to have those conversations around uncertainty and around patient care, those are often the most helpful and like educational, at the bedside, in the moment, day-to-day conversations. Because it's very applicable to cases being discussed in real time” (Cardio 8)

### Resident preferences about how supervisors respond to uncertainty

Although residents were reluctant to express uncertainty to supervisors, residents wanted supervisors to be forthcoming with uncertainty. They viewed supervisors expressing uncertainty as a positive response, and valued the leadership taken to express uncertainty to residents:

“And we really appreciate it when [supervisors] show us that uncertainty. I wouldn't even call it vulnerability because it's not vulnerability. In fact, it's the opposite. It's like it's good leadership. But maybe sometimes people see, expressing not knowing as a form of maybe vulnerability or something like that. I think trainees really appreciate when their staff shows that because we're uncertain all the time.” (Cardio 8)

Residents stated that supervisor expressions of uncertainty validate the uncertainty faced by residents:

“ I remember that staff said to me, very senior cardiologist said, “I don't know either.” And it’s very reassuring because it means that there’s not something I'm missing in the puzzle in front of me.”(Cardio 1)

Residents reflected on times when supervisors minimized uncertainty, which subsequently led to residents being confused about the clinical decisions:

“Sometimes I actually don't like when something isn't black and white, it's something you can have an opinion on, but then the [supervisor] makes it seem like it's black and white because then you get kind of confused when you see something different every day.” (OBGYN 3)

Residents also recalled instances where they realized their supervisors were uncertain through the supervisor’s actions rather than direct communication with the team:

Some [supervisors] will do it through their actions…it won’t be clear in the moment that is uncertainty. And then will come back the next day and be like, “I talked to so and so at this time, and this is what we're going to do.” And then you in retrospect realize that we didn't actually know the answer yesterday, because we've now changed course immediately...” (Cardio 4)

However, residents qualified that they would not want their supervisors to express uncertainty directly in high-stakes situations:

“It’s reassuring in that sense because I'm like OK, I'm also not missing something, right. So if we were in clinic and there was something that you hadn’t seen before and I also hadn’t seen it, I would be like OK, this is cool. This is interesting. We’re both going to learn. But I think in the delivery room, when it comes to making the diagnosis and when it comes to do we have to [perform an urgent C-section on] this person, is this baby going to live or not, or is this mother going to deteriorate and possibly go to [the intensive care unit] it’s not reassuring that my staff also doesn’t know what to do.” (OBGYN 1)

## Discussion

This study provides insights into the way residents perceive and respond to uncertainty in multidisciplinary team environments. We found that residents recognized that uncertainty is unavoidable in clinical medicine. While residents had positive responses in expressing uncertainty to their interdisciplinary peers, they had predominantly negative responses in expressing uncertainty to their supervisors, despite acknowledging positive responses were possible.

Our study adds to the understanding of how residents navigate uncertainty. Although the residents in our study expressed tolerance for uncertainty, additional situational characteristics influenced whether they expressed their uncertainty. Within the same simulation scenario, residents were willing to express uncertainty to peers and reluctant to express uncertainty to supervisors. The positive emotional responses residents frequently described when expressing uncertainty to peers, including comfort, were not held in response to expressing uncertainty to supervisors. This highlights how uncertainty tolerance and expressing uncertainty can be distinct, emphasizing that uncertainty tolerance is more than a state or trait and is moderated by situational characteristics.

Our findings highlight how residents consider the risks and benefits to both themselves and the patient when responding to uncertainty. Aligning with existing literature, our study identified risks such as perceived negative assessments from supervisors (5), perceived negative effects on trust with supervisors (5), and failure to demonstrate the independence residents feel they need to manifest (8). By contrast, the benefits of expressing uncertainty identified by our participants included ensuring patient safety and receiving help.

This balance of risks and benefits was also influenced by the individual’s scope of practice. Residents were more likely to express their uncertainty to supervisors if they perceived that the content they were uncertain about was outside of their expected scope of knowledge. This finding might be related to their perception that expressing uncertainty about content within their scope of practice to their supervisor could negatively impact their relationship with the supervisor and their clinical experiences, however would be unlikely to affect their relationship with other residents. In essence, the supervisor within their specialty could be conceptualized as a gatekeeper to the consequences of expressing uncertainty. Importantly, identifying content as out of scope requires the resident to have a sense of what is “in scope”. More junior residents may struggle to recognize what is “in scope” and what is “out of scope” with limited clinical experience.

Taken together, findings from our study highlight an important overall implication that guides strategies to improve how residents respond to uncertainty. All residents shared a perspective that expressing uncertainty to their supervisor could have negative consequences; the exact type of consequence did not matter to the residents. If we are to change this perception, then we need to promote a learning environment where the expression of uncertainty is an expectation of training rather than a perceived major interpersonal risk. As emphasized in other team contexts [6, 18, 19], this requires effort on the part of supervisors to create an environment that fosters and reinforces expressing uncertainty, by emphasizing the ways in which positive responses to uncertainty can enhance patient safety, help resolve the uncertainty, and provide an opportunity for learning. The active role of supervisors to engage in behaviors that demonstrate the ideals of an environment has been proposed to foster communication skills, such as “speaking up” behaviors [19].

Our study aligns with recent literature suggesting a shift beyond a “mastery learning” focus to simulation training, to scenarios that foster the development of “adaptive expertise”. Clarke et al argue [15] that simulation scenarios need to incorporate the ill-defined scenarios that create the complexity, uncertainty, and clinical ambiguity encountered in authentic clinical situations to support effective learning by simulation rather than simulation of learning. They encourage incorporating the complexity and ambiguity of authentic clinical practice through “epistemic fidelity” rather than prototypical cases with clear-cut management plans. Our study design supports the use of simulations with epistemic fidelity to prompt reflection about navigating authentic scenarios. Additionally, our study design of simulation-primed qualitative inquiry further supports the merits of using simulation with deliberate attention to epistemic fidelity to explore team dynamics [19].

We propose that this work lays the foundation to use simulation to further understand how residents respond to uncertainty. Future work could identify situational characteristics beyond other participants and factors relating to pregnant patients that moderate how residents respond to uncertainty. Future studies exploring how residents respond to uncertainty in simulation-based designs could add to our understanding. Involving debriefs with supervisors as full participants may add further insights into supervisor perceptions when residents express uncertainty and insights into the behaviors of supervisors that foster residents expressing uncertainty. Future studies could also explore how debriefing shapes future responses to uncertainty encountered in team contexts and how residents respond to perceived uncertainty with patients and other allied health professionals (midwives, respiratory therapists, and nurses).

There are limitations that merit further discussion in framing the findings of our study. Firstly, we included senior residents. Our findings may have been different if we incorporated junior residents with less sense of what uncertainty was due to knowledge gaps rather than irreducible and what content was “in scope” and what content was “out of scope”. Secondly, the simulation design limited the opportunities for residents to engage in strategies used when encountering clinical uncertainty, such as drafting, cross-checking, and mental rehearsals [6]. This may have changed the way residents perceived or responded to uncertainty. Thirdly, surgical management was not required in our scenarios. Consequently, OBGYN residents may have approached expressing uncertainty differently than if the scenario occurred in a surgical context. Fourthly, our findings represent the trainee perspective. The perspectives of supervisors may provide additional insights. Finally, the simulation-based design enabled us to manufacture multiple elements of uncertainty in a controlled, observational setting. In doing so, we acknowledge that residents may have changed their behavior despite our attempts to ensure they knew the simulation was a learning rather than a performance-oriented exercise. Consequently, residents may have been less likely to express uncertainty if they felt it was a performance-oriented exercise.

In conclusion, we observed residents perceiving uncertainty to have both positive and negative responses within the same simulation scenario despite holding the belief that uncertainty is unavoidable and that expressing uncertainty can improve patient safety and team communication.

## Data Availability

The datasets used and/or analyzed during the current study are available from the corresponding author on reasonable request.

## Abbreviations

OBGYN: Obstetrics and Gynecology
PGY: Postgraduate year
